# Ocular Biometry and Intra Ocular Lens Power among Cataract Patients in Rural Eastern Ethiopia

**DOI:** 10.4314/ejhs.v31i4.17

**Published:** 2021-07

**Authors:** Mandefro Sintayehu Kassa, Girum W Gessesse

**Affiliations:** 1 Assistant Professor of Ophthalmology, Saint Paul Hospital Millennium Medical College, Addis Ababa, Ethiopia; 2 Associate Professor of Ophthalmology, Saint Paul's Hospital Millennium Medical College, Addis Ababa, Ethiopia

**Keywords:** Axial length, Anterior chamber depth, Corneal curvature, Intraocular lens

## Abstract

**Background:**

The objective of the study was to report on the main parameters of ocular biometry and Intraocular lens (IOL)power of patients attending a cataract surgical campaign in Eastern Ethiopia.

**Methods:**

The study was a cross-sectional study on 765 eyes which were eligible for cataract surgery during a mass cataract surgical campaign conducted from April 04 to April 10, 2018 at Bisidimo Hospital, Eastern Ethiopia. Ocular biometric parameters were measured by automated keratorefractometer and Sonomed A-Scan (Model 300AP) using contact applanation method. Multiple linear regression analysis was done to determine association of ocular biometry components with socio demography of the study subjects.

**Results:**

The mean corneal curvature and anterior chamber depth (ACD, measured from corneal epithelium to lens) were found to be 7.61 mm and 2.88mm respectively. The mean axial length was estimated to be 22.98 mm. The mean refractive power of IOL was calculated to be 19.34D. The mean axial length in females was shorter than that of males by 0.24 (P - value = 0.01). The mean ACD in males was also larger than that of females by 0.1 (P - value = 0.001).

**Conclusion:**

This study provided a larger population based normative data on ocular biometry in Ethiopia. The female sex was a strong predictor of small axial length. Increasing age had no effect on axial length but was found to be a stronger predictor of shallow ACD.

## Introduction

Ocular Biometry is an essential part of ophthalmic evaluation of patients. Many eye diseases and conditions can be predicted by looking at the axial length, corneal curvature and anterior chamber depth of the eye. Researchers indicated that long axial length is associated with primary open angle glaucoma (POAG)while short ocular axis and shallow anterior chamber predispose individuals to primary angle-closure glaucoma (PACG) (1,2). In a study done in India patients with POAG were found to have longer axial length and flatter corneas as compared to age matched controls (3). The Singapore Malay Eye Study(SMES) also demonstrated an association between increasing axial length (AL) and POAG thus suggesting axial myopia as a potential risk factor for POAG (4).

Refractive errors are major causes of visual impairment worldwide (5). A good understanding of ocular biometric parameters like axial length is crucial for understanding the risk factors and determinants of ammetropia (6,7,8).

Anterior chamber depth (ACD, measured from corneal epithelium to lens) is an important parameter in the evaluation of the anterior segment of the eye. Central ACD less than 2.5 mm has been regarded as shallow ACD which is a main risk factor for PACG. Measurement of axial ACD has been used in population screening for angle closure (9). In one study done in China the PACG prevalence was 25% when ACD is between 2.1–2.3 mm but the prevalence was 100% when ACD is less than 1.5 mm (10). Aung T et al also reported ACD as the strongest predictor of PACG (11).

Another most important use of ocular biometry is for the calculation of the power of Intraocular lenses implanted during cataract surgery. The quality of cataract surgery is largely dependent on implantation of the accurate power of intraocular lens which is variable for each patient undergoing the surgery. The critical step in ocular biometry to attain the desired post-operative refractive outcome requires standardization of techniques to ensure accurate measurements for correct calculation of required IOL power (12,13).

A-scan ultrasound is the traditional technique for measuring anterior chamber depth, axial length and lens thickness. It involves passing an ultrasonic beam via a transducer through the eye and as this is returned after hitting intraocular structures a trace of ocular spikes is displayed on the monitor from the cornea to the orbital fat(14). Biometry values can be obtained either by contact (applanation), immersion or optical methods. The contact/applanation technique is a widely used method which requires placing an ultrasound probe on the central cornea. This slightly indents the surface leading to various degrees of corneal compression which may introduce errors into the values(15). The immersion A-scan biometry uses a saline filled scleral (Prager) shell between the probe and the eye; it is relatively observer independent. The optical method is a non-contact technique by partial coherence interferometry (PCI) that is highly reproducible, observer-independent and therefore potentially more accurate (16). The immersion and optical methods give comparative results (17).

Globally, many researchers have reported their finding on ocular biometry: for example the Handan eye study, the Beijing eye study in the northern China and the Liwan eye study in the southern China (18,19,20). In Nepal one study reported a mean axial length and IOL power to be 22.68mm and 21.60D respectively (21).

In our Continent there are few studies on ocular Biometry. One study which was done in Nigeria revealed a mean axial length of the study groups to be 21.7mm(22). There are also few data on the average value of the main parameters of ocular biometry in Ethiopia in large population based study. Thus, this research will provide a normative data on the ocular biometry of Ethiopian patients and also will be taken as reference for many patients in Africa. Knowing the average value of the axial length and the average power of the IOL is especially important in a resource limited countries like ours where there is an extreme scarcity of most important Ophthalmic instruments like automatic keratometer and A-scan.

The main objective of the study was to report on the main parameters of ocular biometry which are keratometry, axial length, anterior chamber depth and Intraocular lens power of patients attending a cataract surgical campaign in Eastern Ethiopia.

## Patients and Methods

The study was a cross sectional study on 765 eyes (721 patients) which were eligible for cataract surgery during a mass cataract surgical campaign conducted from April 04 to April 10, 2018 at Bisidimo Hospital, eastern Harargie zone in Eastern Ethiopia. The population comprised of all age groups living in 12 districts of Eastern Harargie Zone. One exclusion criteria is corneal opacity which may have an impact on ocular biometric parameters. Data was collected from the clinical format that is routinely used for recording the name of the patient, age, sex and ethnic group of patients undergoing surgery during campaign.

Ocular biometric parameters such as axial length (AL), anterior chamber depth (ACD), radius of corneal curvature (K) were measured by automated keratorefractometer (Retinomax) and Sonomed A-Scan(Model 300AP) using contact applanation method. Radius of corneal curvature in the vertical and horizontal meridian (K^1^ and K^2^) was initially measured, and the mean corneal curvature radius (MCC) was calculated as the average of the steep and flat curvatures. Power of Intraocular lens needed for each cataract eyes was calculated using SRK-T formula. All measurements were taken by two experienced optometrists. The outcome or dependent variables were axial length, mean corneal curvature, anterior chamber depth and power of Intraocular lens. The independent variables include age, sex and ethnicity of the study groups.

Data was cleaned, edited and entered to SPSS 21.0 Software for analysis. Analysis of variance (ANOVA) was conducted to evaluate the variation in different biometric components. Univariate and multivariate analysis were performed to determine association of ocular biometric components with socio demography of the study participants.

**Ethics approval and consent to participate**: ethical clearance to conduct the study was obtained from the IRB of Bisidimo Hospital. Study participants were also explained about the purpose of the study and verbal consent was obtained. The use of verbal consent to conduct the study was approved by the ethics committee. For participants under 16 years old, informed verbal consent was obtained from their parent or guardian. The collected data and personal identification related to each data were kept secret and confidential.

## Results

A total of 765 eyes were enrolled in this study. The mean age of our study groups was 60.6 ± 14.8 years, with a range of 3 years -100 years. The majority, 441 (57.6%) of the study groups were females and the rest 324 (42.2%) were males. Most of the study participants were greater than 40 years of age. Only Ten percent (10%) of the study groups were below 40 years of age (Table 1).

**Table 1:** Mean values of AL, ACD, MCC and IOL power across different age groups of the study participants at Bisidimo Hospital mass eye campaign, April 2018

AGE GROUP (yrs)		AL (mm)	ACD(mm)	IOL POWER(D)	MCC(mm)
3–40	Mean	23.7162	3.1889	16.7797	7.5861
	N	37	37	37	37
41–60	Mean	22.9056	2.8816	19.2263	7.5843
	N	411	411	411	411
61–80	Mean	22.9525	2.8555	19.8488	7.6595
	N	291	291	291	291
81+	Mean	23.5388	2.9046	19.1462	7.6329
	N	26	26	26	26
Total	Mean	22.9842	2.8873	19.3421	7.6146
	N	765	765	765	765

AL = Axial length; ACD = Anterior chamber depth; MCC = Mean Corneal curvature; IOL POWER = Intraocular lens power

The mean corneal curvature was found to be 7.61 mm (with a 95% CI between 7.58 and 7.64) and the mean anterior chamber depth was estimated to be 2.88 mm (with a 95% CI between 2.86 and 2.91). With regard to axial length the minimum and maximum axial length were found to be 12.85 mm and 32.82 mm respectively and the mean axial length was measured to be 22.98 mm (with a 95% CI between 22.89 and 23.07). All the three important parameters of ocular biometry followed a normal distribution according to the finding of our study (Table 2).

**Table 2:** Mean values with confidence interval of AL, MCC and ACD of Study participants at Bisidimo Hospital mass eye campaign April 2018

Parameters		Statistic	95% Confidence Interval	
			Lower	Upper
AL	N	765	765	765
	Minimum	12.65		
	Maximum	32.82		
	Mean	22.9842	22.8908	23.0706
	Std. Deviation	1.29635	1.10771	1.47273
CC	Mean	7.6146	7.5871	7.6416
	Std. Deviation	.37778	.32574	.43098
	N	765	765	765
ACD	Mean	2.8873	2.8602	2.9187
	Std. Deviation	.40753	.34358	.48656
	N	765	765	765

The Mean refractive power of the Intraocular lens required for these cataract eyes was calculated to be 19.34D (With a 95% CI between 19.06 and 19.63). The mode and median refractive power of the IOL was found to be 20.00. The minimum and maximum power of intraocular lens used was found to be -5D and 30.5D respectively. The 25^th^ and 75^th^ percentiles were also calculated to be 18.50D and 21.00D respectively (Table 3).

**Table 3:** Statistics on IOL power implanted in cataract eyes at Bisidimo Hospital mass eye campaign April 2018

Parameters			95% Confidence Interval	
		IOL power in D	Lower	Upper
Mean		19.3421	19.0633	19.6313
Median		20.0000	19.5000	20.0000
Mode		20.00		
Std. Deviation		3.92566	3.47904	4.30195
Minimum		-5.00		
Maximum		30.50		
Percentiles	5	12.5000	10.5000	14.0000
	10	16.0000	15.0000	16.5000
	15	17.0000	16.5000	17.5000
	20	18.0000	17.5000	18.0000
	25	18.5000	18.0000	18.5000
	30	18.5000	18.5000	19.0000
	35	19.0000	18.6160	19.0000
	40	19.2000	19.0000	19.5000
	50	20.0000	19.5000	20.0000
	60	20.5000	20.0000	20.5000
	75	21.0000	21.0000	21.5000
	80	21.5000	21.5000	22.0000

When we look at the mean values of each ocular parameters (AL, ACD and MCC) and IOL power across different age groups, we observed that people under 40 years of age had longer AL, deeper AC, flatter corneas and required smaller IOL power than people above 60 (Table 1).

The mean axial length in females (22.88±1.28mm) was shorter than that of males (23.12 ± 1.3 mm) by 0.24 and this was statistically significant (P - value = 0.01). Under multiple linear regression model gender had a statistically significant impact on the axial length taking other factors such as age and ethnicity constant with a regression coefficient of β = -0.102 and P - value of 0.005. The mean anterior chamber depth in males (2.94 ± 0.39 mm) was also larger than that of females (2.85 ± 0.42mm) by nearly 0.1 and this was statistically significant (P - value = 0.001). Under multiple linear regression model both age and gender had a statistically significant impact on the anterior chamber depth with a regression coefficient of β1 = - 0.173 and β2 = - 0.147 and P - value of 0.000 and 0.000 respectively. By looking at the regression model , we can say that age of the study groups had more effect on the anterior chamber depth than gender. Anterior chamber depth was also negatively correlated with age of the study groups with a Pearson's correlation coefficient of -0.151 in consistent with the finding in the regression model.

The mean average corneal curvature in males was also larger than that of females by 0.11 and this was statistically significant with a P - value of 0.000. Under multiple linear regression model gender had a statistically significant impact on the corneal curvature taking other factors such as age and ethnicity constant with a regression coefficient of β = -0.140 and P - value of 0.000. There was no a statistically significant difference on the mean IOL power required for male and female patients. However, under multiple linear regression model increasing power of IOL was required with increasing age of the patient. Age of the study groups had a stronger effect on the power of IOL than other independent variables like sex and ethnicity

## Discussion

The mean age of our study groups (60) was comparable with the findings of the studies done in Nepal (21), Nigeria (22) and China (23). The mean Axial length in our study(22.98 mm) is longer than that of the Nepal study (22.68mm) (21) and Nigerian study (21.7mm) (22) but it is nearly similar to the finding from the study done in china (22.80 mm) (23) and smaller than that of westerners (23.65mm) (24). Axial length was slightly positively correlated with age but this was not statistically significant indicating that axial length was not affected by increasing age. This finding was similar with that of the study in Singapore but disagrees with Hoffer's report on the biometry of 7500 cataract eyes (25,26). The female sex was found to be the single predictor of shorter axial length under multivariate analysis in our study. This finding was consistent with several other studies which reported that the female sex was associated with shorter axial length (8,25).

The Anterior chamber depth (2.88mm) in our study groups was found to be shallower than most other studies (4,23,24). Both the female sex and increasing age were found to be statistically significant predictor of shallow anterior chamber under multivariate analysis. Age of the study groups had stronger effect than gender. Our finding on the effect of gender on axial length and anterior chamber was similar with the result of one review paper which has tried to thoroughly study on several literatures of the world(27). Some researchers indicated that sex-related differences in biometry has been attributed to differences in stature between men and women, particularly height, as adjustment for height in multivariate analyses tended to attenuate the association(7,8). In SiMES, however, sex differences in AL and ACD were still significant in multivariate analyses controlling for stature, suggesting that sex may be an independent determinant of axial length (25). Genetic and other factors may account for the differences in biometry in men and women (28).

The mean corneal curvature in our study groups (7.61mm) was higher than the study in china (7.56mm) but lower compared with the Handan Eye Study and lower compared with the Liwan eye study(18,20). Men were found to have a steeper cornea than women in our study similar to the finding in the SiMES study (25). However this finding was different from that of the review done by Hoffer and Savini in which they reported flatter corneas in male than female (27). Corneal curvature was slightly positively correlated with age but it was not statistically significant.

The Mean Refractive power of the Intra ocular lens required for these cataract eyes was calculated to be 19.34 D (With a 95% CI between 19.06 and 19.63 D). This was similar to a study from New Zealand that showed a mean IOL power in Asian eyes of 19.45D (29). It was however smaller than the finding of the study in Pakistan (21.2 D ± 2.35 D) (30) and Nepal (21.60D±1.74) (21).

Since it has been a common trend in resource limited countries like ours (where there is scarcity of A - Scan and Keratometer) to insert the estimated average power of IOL for every cataract eyes , we were interested to know the percentage of patients who could have been subjected to an over plus IOL had we not done the IOL power calculation. We calculated the percentiles and we found that five percent (5%) of the study participants required an IOL power of less than or equal to 12. we also found that five percent (5%) of the study participants required an IOL power greater than 24. This means that there was a 5% chance of inducing a greater than or equal to 7D refractive error (myopia) and there was also another 5% chance of inducing a greater than or equal to 5D refractive error (Myopia). Overall 10% of all the operated cataract eyes could have developed a greater than or equal to 6D refractive error if IOL power calculation had not been done in this mass eye campaign.

This study is not without limitation: first we used A- scan ultrasound for measurement of the parameters whereas most other studies which we made the comparison used the IOL Master. Second we did not grade the density of the cataract. We also did not measure the height and weight of patients which have been shown by some studies to have an impact on the three important parameters of ocular biometry.

In conclusion, this study is the first of its kind to provide a larger population-based normative data on the most important parameters of ocular biometry (AL,ACD &MCC) in Ethiopia which can also be used as a substantial reference for African patients. Axial length of our patients was longer than that of patients in Nigeria but was smaller than that of study groups in the west and china. The female sex was a strong predictor of small axial length and shallow anterior chamber. Increasing age had no effect on the axial length but was found to be a stronger predictor of shallow anterior even more than the female sex.
